# Supplementary material for: GGDEF domain as spatial on-switch for a phosphodiesterase by interaction with landmark protein HubP
Source: NPJ Biofilms Microbiomes. 2022 May 2;8:35. doi: 10.1038/s41522-022-00297-w (PMC9061725; doi:10.1038/s41522-022-00297-w)
Supplement: Supplementary file 1 — Supplementary Material [file 41522_2022_297_MOESM1_ESM.pdf]

GGDEF domain as spatial on-switch for a phosphodiesterase by interaction with landmark protein HubP

- SUPPLEMENTARY INFORMATION -

Tim Rick[1], Vanessa Kreiling[1], Alexander Höing[2], Svenja Fiedler[3,4], Timo Glatter[5], Wieland Steinchen[3,4], Georg Hochberg[6], Heike Bähre[7], Roland Seifert[7], Gert Bange[3,4], Shirley Knauer[2], Peter L. Graumann[3,4], Kai M. Thormann[1]

[1]Justus-Liebig-Universität, Department of Microbiology and Molecular Biology, 35392 Giessen, Germany

[2]University Duisburg-Essen, Faculty of Biology, 45141 Essen, Germany

[3]LOEWE Center for Synthetic Microbiology, SYNMIKRO, 35043 Marburg, Germany

[4]Philipps-Universität Marburg, Department of Chemistry, 35043 Marburg, Marburg, Germany

[5]Max Planck Institute for Terrestrial Microbiology, Facility for Mass Spectrometry and Proteomics 35043 Marburg, Germany

[6]Max Planck Institute for Terrestrial Microbiology, 35043 Marburg, Germany

[7]Medizinische Hochschule Hannover, ZFA Metabolomics, 30625 Hannover, Germany

Contents:

Supplementary Tables 1 – 3

Supplementary Figures 1 – 12

Supplementary Table 1: Bacterial strains that were used in this study

Strain	Genotype	Purpose	Reference
Escherichia coli			
**DH5α λpir**	φ80d*lacZ* ΔM15 Δ(*lacZYA-argF*)U169 *recA*$_1$ *hsdR17 deoR thi-l supE44 gyrA96 relA*$_1$*/λpir*	cloning strain	[1]
**WM3064**	*thrB1004 pro thi rpsL hsdS lacZ* ΔM15 RP4-1360 Δ(*araBAD*) 567Δ*dapA* 1341::[*erm pir*(wt)]	conjugation strain for *Shewanella*	[2]
**BL21(DE3)**	*fhuA2* [*lon*] *ompT gal* (λ *DE3*) [*dcm*] Δ*hsdS* λ *DE3* = λ *sBamHIo* Δ*EcoRI-B int*::(*lacI*::*PlacUV5*::*T7 gene1*) *i21* Δ*nin5*	protein overproduction strain	[3]
***Shewanella putrefaciens* CN-32**			
**S757**	CN-32 wt	wildtype strain	[4]
**S2576**	Δ*flaAB*$_2$	markerless in-frame deletion of the lateral flagellins (*sputcn32_3455-3456*)	[5]
**S3297**	Δ*pdeB*	markerless in-frame deletion of the gene *sputcn32_3405 (pdeB)*	[6]
**S4234**	*pdeB-sfgfp*	markerless in-frame fusion of *pdeB* with sf*gfp*	[6]
**S4237**	*pdeB*-E637A-*sfgfp*	functional markerless substitution of the EIL motif to AIL (residue 637) in the background of *pdeB-sfgfp*	[6]
**S4357**	Δ*flaAB*$_2$ Δ*pdeB*	markerless in-frame deletion of the gene *pdeB* (*sputcn32_3405*) in a background with deleted lateral flagellins	[6]
**S6452**	*pdeB-gfp* V522G V523G Q524G	markerless in-frame fusion of *pdeB* with sf*gfp* substitution of Q524G	this study
**S6453**	*pdeB-gfp* K490G V491G M492G Q593G	markerless in-frame fusion of *pdeB* with sf*gfp* substitution of M492G Q593G	this study
**S6454**	*pdeB-GFP* R557G A558G P559G Y560G	markerless in-frame fusion of *pdeB* with *sfgfp* substitution of A558G P559G Y560G	this study
**S6496**	Δ*flaAB*$_2$ Δ*mshE*	markerless in-frame deletion of the gene *mshE (sputcn32_0563)* in a background with deleted lateral flagellins	this study
**S6497**	Δ*flaAB*$_2$ Δ*pdeB* Δ*mshE*	markerless in-frame deletion of the gene *mshE (sputcn32_0563)* in a background with deleted lateral flagellins and deletion of the gene *pdeB* (*sputcn32_3405*)	this study
**S6527**	*pdeB-gfp* K490D Q493A	markerless in-frame fusion of *pdeB* with sf*gfp* substitution of K490D Q493A	this study
**S6528**	*pdeB-gfp* Q524A K527D Q528A	markerless in-frame fusion of *pdeB* with sf*gfp* substitution of Q524A K527D Q528A	this study
**S6659**	*pdeB-gfp* Q524S Q528S	markerless in-frame fusion of *pdeB* with sf*gfp* substitution of Q524S Q528S	this study
**S6683**	Δ*flaAB*$_2$ *mshA* S68C	functional substitution of *mshA* S68C in a background with deleted lateral flagellins	this study
**S6684**	Δ*flaAB*$_2$ Δ*pdeB* *mshA* S68C	functional substitution of *mshA* S68C in a background with deleted lateral flagellins and deletion of the gene *pdeB* (*sputcn32_3405*)	this study

**S6688**	Δ*flaAB*$_2$ Δ*aggA*	markerless in-frame deletion of the gene *aggA (sputcn32_3594)* in a background with deleted lateral flagellins	this study
**S6689**	Δ*flaAB*$_2$ Δ*pdeB* Δ*aggA*	markerless in-frame deletion of the gene *aggA (sputcn32_3594)* in a background with deleted lateral flagellins and deletion of the gene *pdeB (sputcn32_3405)*	this study
**S6690**	Δ*flaAB*$_2$ Δ*pdeB* Δ*mshE* Δ*aggA*	markerless in-frame deletion of the gene *aggA (sputcn32_3594)* in a background with deleted lateral flagellins, and deletion of the genes *pdeB (sputcn32_3405)* and *mshE (sputcn32_0563)*	this study
**S6691**	Δ*flaAB*$_2$ Δ*pilB*	markerless in-frame deletion of the gene *pilB (sputcn32_3423)* in a background with deleted lateral flagellins	this study
**S6692**	Δ*flaAB*$_2$ Δ*pdeB* Δ*pilB*	markerless in-frame deletion of the gene *pilB (sputcn32_3423)* in a background with deleted lateral flagellins and deletion of the gene *pdeB (sputcn32_3405)*	this study
**S6693**	Δ*flaAB*$_2$ Δ*pdeB* Δ*mshE* Δ*pilB*	markerless in-frame deletion of the gene *pilB (sputcn32_3423)* in a background with deleted lateral flagellins, and deletion of the genes *pdeB (sputcn32_3405)* and *mshE (sputcn32_0563)*	this study
**S6729**	*pdeB-gfp* K527E Q528S	markerless in-frame fusion of *pdeB* with sf*gfp* substitution of K527E Q528S	this study
**S7024**	*pdeB-mvenus*	markerless in-frame fusion of *pdeB* with *mvenus*	this study
**S7025**	*pdeB-mvenus* D508A E509A	markerless in-frame fusion of *pdeB* D508A E509A with *mvenus*	this study
**S7026**	*pdeB-mvenus* E637A	markerless in-frame fusion of *pdeB* E637A with *mvenus*	this study
**S7243**	*pdeB-gfp* K527S Q528S	markerless in-frame fusion of *pdeB* with sf*gfp* substitution of K527S Q528S	this study
**S7244**	*pdeB-gfp* K527S	markerless in-frame fusion of *pdeB* with *sfgfp* substitution of K527S	this study
**S7245**	*pdeB-gfp* G497A	markerless in-frame fusion of *pdeB* with *sfgfp* substitution of G497A	this study
**S7246**	*pdeB-gfp Q499S*	markerless in-frame fusion of *pdeB* with *sfgfp* substitution of Q499S	this study
**S7247**	*pdeB-gfp* E500S	markerless in-frame fusion of *pdeB* with *sfgfp* substitution of E500S	this study
**S7444**	Δ*flaAB*$_2$ Δ*mshE* Δ*aggA*	markerless in-frame deletion of the gene *aggA (sputcn32_3594)* in a background with deleted lateral flagellins and deletion of the gene *mshE (sputcn32_0563)*	this study
**S7445**	Δ*flaAB*$_2$ Δ*mshE* Δ*pilB*	markerless in-frame deletion of the gene *pilB (sputcn32_3423)* in a background with deleted lateral flagellins and deletion of the gene *mshE (sputcn32_0563)*	this study
**S7504**	*pdeB* K527E Q528S	markerless in-frame substitution of *pdeB* K527E Q528S	this study
**S7505**	*pdeB* G497A	markerless in-frame substitution of *pdeB* G497A	this study
**S7506**	*pdeB* K578S	markerless in-frame substitution of *pdeB* K578S	this study

**S7507**	*pdeB-mvenus* K527E Q528S	markerless in-frame fusion of *pdeB*-K527E Q528S with *mvenus*	this study
**S7508**	*pdeB* K580S	markerless in-frame substitution of *pdeB* K580S	this study
**S7562**	*pdeB-gfp* K578S	markerless in-frame fusion of *pdeB* with *sfgfp* substitution of K578S	this study
**S7564**	*pdeB-gfp* K580S	markerless in-frame fusion of *pdeB* with *sfgfp* substitution of K580S	this study
**S7614**	pMMB-Gm-Bc3-5 AAV (hok-sok)	wildtype strain containing the c-di-GMP biosensor plasmid	[7]
**S7616**	Δ*pdeB* pMMB-Gm-Bc3-5 AAV (hok-sok)	c-di-GMP biosensor plasmid in the background of deleted *pdeB* (*sputcn32_3405*)	[7]
**S7653**	*pdeB* K527E Q528S pMMB-Gm-Bc3-5 AAV (hok-sok)	c-di-GMP biosensor plasmid in the background of *pdeB* K527E Q528S substitution	[7]
**S7654**	*pdeB* G497A pMMB-Gm-Bc3-5 AAV (hok-sok)	c-di-GMP biosensor plasmid in the background of *pdeB* G497A substitution	[7]
**S7655**	*pdeB* K578S pMMB-Gm-Bc3-5 AAV (hok-sok)	c-di-GMP biosensor plasmid in the background of *pdeB* K578S substitution	[7]
**S7691**	*lapA*-GS-3xFLAG	functional markerless in-frame tag of 3xFLAG to the C-terminus of *lapA* via a flexible GS-linker	this study
**S7692**	Δ*pdeB lapA*-GS-3xFLAG	functional markerless in-frame tag of 3xFLAG to the C-terminus of *lapA* via a flexible GS-linker in the background of deleted *pdeB (sputcn32_3405)*	this study
**S7703**	*lapB*-GS-3xFLAG	functional markerless in-frame tag of 3xFLAG to the C-terminus of *lapB* via a flexible GS-linker	this study
**S7704**	Δ*pdeB lapB*-GS-3xFLAG	functional markerless in-frame tag of 3xFLAG to the C-terminus of *lapB* via a flexible GS-linker in the background of deleted *pdeB (sputcn32_3405)*	this study
***Shewanella oneidensis* MR-1 strains**			
**S79**	MR-1 wt	wildtype strain	[8]
**S7296**	Δ*pdeB*	Markerless in-frame deletion of *pdeB* of *S. oneidensis* MR-1	this study
**S7294**	*pdeB-gfp*	Markerless in-frame fusion of *pdeB* with *sfgfp* in *S. oneidensis* MR-1	this study
**S7423**	pMMB-Gm-Bc3-5 AAV (hok-sok)	MR-1 wildtype strain containing the c-di-GMP biosensor plasmid	[7]
**S7425**	Δ*pdeB* pMMB-HS-Bc-3-5-AAV (hok-sok)	c-di-GMP biosensor plasmid in the background of deleted *pdeB* (*SO_0437*)	[7]

Supplementary Table 2: Plasmids that were used in this study

Plasmid	Relevant genotype or phenotype	Reference
pNPTS-138-R6KT	*mob*RP4+ *ori*-R6K *sacB* β-galactosidase fragment alpha, suicide plasmid for in frame deletions/insertions in *Shewanella*, Km^r	[9]
pET-24c	overproduction vector for His-tagged proteins	[10]
pBTOK	pBBR1-MCS2 backbone (pBBR origin, Km^r); TetR, Promoter and multiple cloning site of pASK-IBA3plus and *E. coli* rrnB1 T1 and lambda phage T0 terminator. Overproduction plasmid, inducible with anhydrotetracycline	[11]
pMMB-Gm-Bc3-5 AAV (hok-sok)	pMMB67EH (Gm) backbone containing the c-di-GMP biosensor (turboRFP with an AAV tag) and also the hok/sok region from pXB300. Used as c-di-GMP reporter.	[7]
**overexpression vectors**		
pET24c MBP-PdeB (MR-1) GGDEF-6xHis	Vector used to express the GGDEF-domain of MR-1 PdeB (residues 417 - 585) with N-terminal MBP and C-terminal 6xHis translational fusion	this study
pET24c MBP-PdeB (MR-1) GGDEF-6xHis K524S	Vector used to express the GGDEF-domain of MR-1 PdeB (residues 417 - 585) with N-terminal MBP and C-terminal 6xHis translational fusion	this study
pET24c MBP-PdeB (MR-1) GGDEF-6xHis Q525S	Vector used to express the GGDEF-domain of MR-1 PdeB (residues 417 - 585) with N-terminal MBP and C-terminal 6xHis translational fusion	this study
pET24c MBP-PdeB (MR-1) GGDEF-6xHis K524E Q525S	Vector used to express the GGDEF-domain of MR-1 PdeB (residues 417 - 585) with N-terminal MBP and C-terminal 6xHis translational fusion	this study
pET24c MBP-PdeB (MR-1) GGDEF-6xHis G494A	Vector used to express the GGDEF-domain of MR-1 PdeB (residues 417 - 585) with N-terminal MBP and C-terminal 6xHis translational fusion	this study
pET24c MBP-PdeB (MR-1) GGDEF-6xHis E497S	Vector used to express the GGDEF-domain of MR-1 PdeB (residues 417 - 585) with N-terminal MBP and C-terminal 6xHis translational fusion	this study
pET24c MBP-PdeB (MR-1) PAS-GGDEF-6xHis	Vector used to express the PAS- and GGDEF-domain of MR-1 PdeB (residues 304 - 585) with N-terminal MBP and C-terminal 6xHis translational fusion	this study
pET24c MBP-PdeB (MR-1) PAS-GGDEF-6xHis K524E Q525S	Vector used to express the PAS- and GGDEF-domain of MR-1 PdeB (residues 304 - 585) with N-terminal MBP and C-terminal 6xHis translational fusion	this study
pET24c (MR-1) FimV-Cdomain-6xHis	Vector used to express the C-terminal domain of MR-1 FimV (residues 1000 - 1110) with C-terminal 6xHis translational fusion	this study
pET24c 3xFLAG-(CN-32) HubP-FimV-Cdomain-6xHis	Vector used to express the C-terminal domain of CN-32 FimV (residues 1000 - 1110) with C-terminal 6xHis translational fusion and 3xFLAG	this study
pET24c MBP-PdeB (CN-32) GGDEF-EAL-6xHis	Vector used to express the GGDEF- and EAL-domain of CN-32 PdeB (residues 420-847) with N-terminal MBP and C-terminal 6xHis translational fusion	this study
pET24c *mshE*-6xHis	Vector used to express MshE of CN-32 with C-terminal 6xHis translational fusion	this study
pET24c *mshE*_Ndomain-6xHis	Vector used to express the N-terminal domain of CN-32 MshE (residues 2 - 145) with C-terminal 6xHis translational fusion	this study
pET24c *pilB*_Ndomain-6xHis	Vector used to express the N-terminal domain of CN-32 PilB (residues 2 - 145) with C-terminal 6xHis translational fusion	this study
pBTOK *dgcA*-6xHis	Vector for ectopical expression of *dgcA* (*E. coli*) in *S. putrefaciens* CN-32 with C-terminal 6xHis	this study
pBTOK *dgcA*-6xHis D216E	Vector for ectopical expression of *dgcA* (*E. coli*) in *S. putrefaciens* CN-32 with C-terminal 6xHis and D216E	this study

pBTOK *dgcA*-6xHis E276K	Vector for ectopical expression of *dgcA* (*E. coli*) in *S. putrefaciens* CN-32 with C-terminal 6xHis and E276K	this study
**In-frame insertion vectors**		
pNPTS CN-32 *pdeB-gfp* K527S	Suicide vector for markerless in-frame insertion of *pdeB-gfp* of *S. putrefaciens* CN-32 with K527S	this study
pNPTS CN-32 *pdeB-gfp* K527S Q528S	Suicide vector for markerless in-frame insertion of *pdeB-gfp* of *S. putrefaciens* CN-32 with K527S Q528S	this study
pNPTS CN-32 *pdeB-gfp* K527D	Suicide vector for markerless in-frame insertion of *pdeB-gfp* of *S. putrefaciens* CN-32 with K527D	this study
pNPTS CN-32 *pdeB-gfp* K527D Q528S	Suicide vector for markerless in-frame insertion of *pdeB-gfp* of *S. putrefaciens* CN-32 with K527D Q528S	this study
pNPTS CN-32 *pdeB-gfp* Q524A K527D Q528A	Suicide vector for markerless in-frame insertion of *pdeB-gfp* of *S. putrefaciens* CN-32 with Q524A K527D Q528A	this study
pNPTS CN-32 *pdeB-gfp* G497A	Suicide vector for markerless in-frame insertion of *pdeB-gfp* of *S. putrefaciens* CN-32 with G497A	this study
pNPTS CN-32 *pdeB-gfp* Q499S	Suicide vector for markerless in-frame insertion of *pdeB-gfp* of *S. putrefaciens* CN-32 with Q499S	this study
pNPTS CN-32 *pdeB-gfp* E500S	Suicide vector for markerless in-frame insertion of *pdeB-gfp* of *S. putrefaciens* CN-32 with E500S	this study
pNPTS CN-32 *pdeB-gfp* Q528S	Suicide vector for markerless in-frame insertion of *pdeB-gfp* of *S. putrefaciens* CN-32 with Q528S	this study
pNPTS CN-32 *pdeB-gfp* Q524S	Suicide vector for markerless in-frame insertion of *pdeB-gfp* of *S. putrefaciens* CN-32 with Q524S	this study
pNPTS CN-32 *pdeB-gfp* Q524S Q528S	Suicide vector for markerless in-frame insertion of *pdeB-gfp* of *S. putrefaciens* CN-32 with Q524S Q528S	this study
pNPTS CN-32 *pdeB-gfp* K527E Q528S	Suicide vector for markerless in-frame insertion of *pdeB-gfp* of *S. putrefaciens* CN-32 with K527E Q528S	this study
pNPTS CN-32 *pdeB-gfp* K490D Q493A	Suicide vector for markerless in-frame insertion of *pdeB-gfp* of *S. putrefaciens* CN-32 with K490D Q493A	this study
pNPTS CN-32 *pdeB-gfp* R557G A558G P559G Y560G	Suicide vector for markerless in-frame insertion of *pdeB-gfp* of *S. putrefaciens* CN-32 with R557G A558G P559G Y560G	this study
pNPTS CN-32 *pdeB-gfp* V522G V523G Q524G	Suicide vector for markerless in-frame insertion of *pdeB-gfp* of *S. putrefaciens* CN-32 with V522G V523G Q524G	this study
pNPTS CN-32 *pdeB-gfp* K490G V491G M492G Q593G	Suicide vector for markerless in-frame insertion of *pdeB-gfp* of *S. putrefaciens* CN-32 with K490G V491G M492G Q593G	this study
pNPTS CN-32 *pdeB-gfp* K578S	Suicide vector for markerless in-frame insertion of *pdeB-gfp* of *S. putrefaciens* CN-32 with K578S	this study
pNPTS CN-32 *pdeB-gfp* K580S	Suicide vector for markerless in-frame insertion of *pdeB-gfp* of *S. putrefaciens* CN-32 with K580S	this study
pNPTS CN-32 *pdeB* G497A	Suicide vector for markerless in-frame insertion of *pdeB* of *S. putrefaciens* CN-32 with G497A	this study
pNPTS CN-32 *pdeB* K527E Q528S	Suicide vector for markerless in-frame insertion of *pdeB* of *S. putrefaciens* CN-32 with K527E Q528S	this study
pNPTS CN-32 *pdeB* K578S	Suicide vector for markerless in-frame insertion of *pdeB* of *S. putrefaciens* CN-32 with K578S	this study
pNPTS CN-32 *pdeB* K580S	Suicide vector for markerless in-frame insertion of *pdeB* of *S. putrefaciens* CN-32 with K580S	this study
pNPTS CN-32 *pdeB-venus*	Suicide vector for markerless in-frame insertion of *pdeB-mvenus* of *S. putrefaciens* CN-32	this study
pNPTS CN-32 *pdeB-venus* D508A E509A	Suicide vector for markerless in-frame insertion of *pdeB-mvenus* of *S. putrefaciens* CN-32 with 508A E509A	this study
pNPTS CN-32 *pdeB-venus* E637A	Suicide vector for markerless in-frame insertion of *pdeB-mvenus* of *S. putrefaciens* CN-32 with E637A	this study
pNPTS CN-32 *lapA*-GS-3xFLAG	Suicide vector for markerless in-frame insertion of 3xFLAG to the C-terminus of *lapA* via a flexible GS-linker	this study
pNPTS CN-32 *lapB*-GS-3xFLAG	Suicide vector for markerless in-frame insertion of 3xFLAG to the C-terminus of *lapB* via a flexible GS-linker	this study
pNPTS CN-32 *mshA* S68C	Suicide vector for markerless in-frame insertion of *mshA* of *S. putrefaciens* CN-32 with S68C	this study

pNPTS MR-1 *pdeB-gfp*	Suicide vector for markerless in-frame insertion of *pdeB-gfp* of *S. oneidensis* MR-1	this study
**In-frame deletion vectors**		
pNPTS CN-32 Δ*mshE*	Suicide vector for markerless in-frame deletion of *mshE* of *S. putrefaciens* CN-32	this study
pNPTS CN-32 Δ*aggA*	Suicide vector for markerless in-frame deletion of *aggA* of *S. putrefaciens* CN-32	this study
pNPTS CN-32 Δ*pilB*	Suicide vector for markerless in-frame deletion of *pilB* of *S. putrefaciens* CN-32	this study
pNPTS MR-1 Δ*pdeB*	Suicide vector for markerless in-frame deletion of *pdeB* of *S. oneidensis* MR-1	this study

Supplementary Table 3: Primer that were used in this study

Plasmid	Primer	Sequence
pET24c MBP-PdeB (MR-1) GGDEF-6xHis	TR258 MBP fw	TTAACTTTAAGAAGGAGATATACAATGAAAATAGAAGAAGGTAAACTGGTAATCTGG
	TR259 MBP rv	GCTGCCCCCGAGGTTGTTGTTATTGTTATTGT
	TR260	AATAACAACAACCTCGGGGGCAGCGAAGAACTTCTTAAGCATCAGCTAC
	TR257	GTGGTGGTGGTGGTGGTGCTCAATGGTGATGGTGATGGTGGTAAATGTGGATTTGGTTGGTGC
pET24c MBP-PdeB (MR-1) GGDEF-6xHis K524S	TR510 MBP ol plas fw	TTAACTTTAAGAAGGAGATATACAATGAAAATAGAAGAAGGTAAACTGGTAATCTGG
	TR511 So KtoS fw	CAATAATTTGGCTCAGCAACTGCGCCACAGC
	TR512 So KtoS rv	GTTGCTGAGCCAAATTATTGCTCAAGTATCGCTGC
	TR513 soGGDEF ol plas rv	GTGGTGGTGGTGGTGGTGCTCAATGGTGATGGTGATGGTGGTAAATGTGGATTTGGTTGGTGC
pET24c MBP-PdeB (MR-1) GGDEF-6xHis Q525S	TR510 MBP ol plas fw	TTAACTTTAAGAAGGAGATATACAATGAAAATAGAAGAAGGTAAACTGGTAATCTGG
	TR514 So QtoS fw	GAGCAATAATGCTCTTCAGCAACTGCGCCACAGC
	TR515 So QtoS rv	GCTGAAGAGCATTATTGCTCAAGTATCGCTGC
	TR513 soGGDEF ol plas rv	GTGGTGGTGGTGGTGGTGCTCAATGGTGATGGTGATGGTGGTAAATGTGGATTTGGTTGGTGC
pET24c MBP-PdeB (MR-1) GGDEF-6xHis K524E Q525S	TR510 MBP ol plas fw	TTAACTTTAAGAAGGAGATATACAATGAAAATAGAAGAAGGTAAACTGGTAATCTGG
	TR593 SO KQ to ES rv	TAATGCTTTCCAGCAACTGCGCCACAGCTAA
	TR594 SO KQ to ES fw	GCAGTTGCTGGAAAGCATTATTGCTCAAGTATCGCTGCAAGTG
	TR513 soGGDEF ol plas rv	GTGGTGGTGGTGGTGGTGCTCAATGGTGATGGTGATGGTGGTAAATGTGGATTTGGTTGGTGC

pET24c MBP-PdeB (MR-1) GGDEF-6xHis G494A	TR510 MBP ol plas fw	TTAACTTTAAGAAGGAGATATACAATGAAAATAGAAGAAGGTAAACTGGTAATCTGG
	TR544 G494A fw	ATTCCTGTGGCGCAAGACATGACTGAATCGCCCTAG
	TR555 G494A rv	ATGTCTTGCGCCACAGGAATTATTAGCCCGCA
	TR513 soGGDEF ol plas rv	GTGGTGGTGGTGGTGGTGCTCAATGGTGATGGTGATGGTGGTAAATGTGGATTTGGTTGGTGC
pET24c MBP-PdeB (MR-1) GGDEF-6xHis E497S	TR510 MBP ol plas fw	TTAACTTTAAGAAGGAGATATACAATGAAAATAGAAGAAGGTAAACTGGTAATCTGG
	TR558 E497S fw	GGGCTAATAACGACTGTGGCCCAAGACATGACT
	TR559 E497S rv	GCCACAGTCGTTATTAGCCCGCATAGGAGGTG
	TR513 soGGDEF ol plas rv	GTGGTGGTGGTGGTGGTGCTCAATGGTGATGGTGATGGTGGTAAATGTGGATTTGGTTGGTGC
pET24c MBP-PdeB (MR-1) PAS-GGDEF-6xHis	TR588 MBP fw SO	TTAACTTTAAGAAGGAGATATACAATGAAAATAGAAGAAGGTAAACTGGTAATCTGG
	TR589 MBP rv OL SO	TACCGCGCTCCCCGAGGTTGTTGTTATTGTTATTGT
	TR590 pet SO PAS fw	CAACCTCGGGGAGCGCGGTAAAATAACCTTAGA
	TR591 pet rv	GTGGTGGTGGTGGTGGTGCTCAATGGTGATGGTGATGGTGGTAAATGTGGATTTGGTTGGTGC
pET24c MBP-PdeB (CN-32) GGDEF-EAL-6xHis	TR258 MBP fw	TTAACTTTAAGAAGGAGATATACAATGAAAATAGAAGAAGGTAAACTGGTAATCTGG
	TR259 MBP rv	GCTGCCCCCGAGGTTGTTGTTATTGTTATTGT
	TR616 rvsn SpGGDEF fw	AATAACAACAACCTCGGGGGCAGCATTACTCAAGAAGAGTTACTGAAGCG
	TR617 rvsn SpEAL rv	GTGGTGGTGGTGGTGGTGCTCAATGGTGATGGTGATGGTGTGCGCGTTGTGCTAAACCCAT
pET24c mshE-6xHis	TR258 MshE OW fw	TTAACTTTAAGAAGGAGATATACAATGAAACCCAGATTAAAGATGCGTTT
	TR259 MshE OW rv	GTGGTGGTGGTGGTGGTGCTCAATGGTGATGGTGATGGTGCGCCTCAACGCCTTGTTGG
pET24c mshE_Ndomain-6xHis	TR372	TTAACTTTAAGAAGGAGATATACAATGCACCATCACCATCACCATAAACCCAGATTAAAGATGCGTTTGG
	TR383	GTGGTGGTGGTGGTGGTGCCTAACGACGATAAAGATTATCAAAGGCC
pET24c pilB_Ndomain-6xHis	TR374	TTAACTTTAAGAAGGAGATATACAATGCACCATCACCATCACCATATGCCAACCACTGGTCTTCATTTA
	TR384	GTGGTGGTGGTGGTGGTGCCTATTCAAGGATTTTTTCAAGGGCTTTAG
pNPTS CN-32 Δ*aggA*	TR377	GCGAATTCGTGGATCCAGATTGAAATCAGCCCTAGACGAAGC
	TR378	TGTTAGTTCCTACTAAAGTATTCATTGCAAACCTCC
	TR379	TACTTTAGTAGGAACTAACAAATGAAAACCGTAATC

	TR380	GCCAAGCTTCTCTGCAGGATGGAGTTTGTTCTAATACTATTGGGC
pNPTS CN-32 Δ*pilB*	TR320 PilB KO1	GAATTCGTGGATCCAGATATGTATAAGCTGGAGATAAATATGAAAGG
	TR321 PilB KO2	TCGTCACCCGACCAGTGGTTGGCATAGATTCTTAA
	TR322 PilB KO3	AACCACTGGTCGGGTGACGAGTTTTTAACAGC
	TR323 PilB KO4	CAAGCTTCTCTGCAGGATCTTTTGGGCTCAATCTTCTTTGG
pNPTS CN-32 Δ*mshE*	AP241 EcoRV 0563 up fw	GAATTCGTGGATCCAGATGCTTACGCCAAGCCAGCTC
	AP242 OL_0563_up_rv	CCTCAACGCCCATCTTTAATCTGGGTTTCATTGGC
	AP243 OL_0563_down_fw	ATTAAAGATGGGCGTTGAGGCGTAATTATGC
	AP244 EcoRV_0563_down_rv	CAAGCTTCTCTGCAGGATCAAGGCAAATCGGCACCAAAG
pNPTS CN-32 *mshA* S68C	TR353	GCGAATTCGTGGATCCAGATAAATGTAACCGACGACGCACAG
	TR357	ATACATCCTTACACTCCACACCCTGAATAGCCG
	TR358	GGGTGTGGAGTGTAAGGATGTATCTAGCATTATTATCGATG
	TR356	GCCAAGCTTCTCTGCAGGATGCTAGGCAGGCCTTTTCTAGTA
pNPTS CN-32 *lapA*-GS-3x-FLAG	VK239 EcoRV OL up 3591 fw 2	GCGAATTCGTGGATCCAGATGGTGGTAGCCACAACGATGC
	VK240 up 3591 OL FLAG rv	AATATCATGATCTTTATAATCGCCATCATGATCTTTATAATCACTGCCAGGGATCATAGTGCCATTGTTATGAG
	VK241 OL FLAG 3591 down fw	GGCGATTATAAAGATCATGATATTGATTATAAAGATGATGATGATAAATAAATAAAATCGTTTTGATGGCCTATAGAAATATAGG
	VK233 EcoRV 3591-down rv	GCCAAGCTTCTCTGCAGGATGGCTTCTAGTGACTCAATATTGAGTGTC
pNPTS CN-32 *lapB*-GS-3x-FLAG	VK222 EcoRV OL up 3592 fw	GCGAATTCGTGGATCCAGATCCCTAGCGATCTACGCCG
	VK223 up 3592 OL FLAG rv	AATATCATGATCTTTATAATCGCCATCATGATCTTTATAATCACTGCCTTTTTTACTGCCCCCATTGAACAG
	VK224 OL FLAG 3592 down fw	GATTATAAAGATCATGATATTGATTATAAAGATGATGATGATAAATAGGTTCAATGGGGGCAGTAAAAAATG
	VK225 EcoRV 3592 down rv	GCCAAGCTTCTCTGCAGGATCTCCGCCGCCACTATACTATC
pNPTS CN-32 *pdeB-gfp* K527S	TR564 pdeb OL plas fw	GCCAAGCTTCTCTGCAGGATGCAAGGCAATATGGATCCATCC
	TR568 K to S rv	TGATCTGGCTTAACAACTGCACCACAGATAAAGC
	TR579 K to S fw	GCAGTTGTTAAGCCAGATCAGTGCTCAAGTCTCATTACAAG
	TR567 pdeB OL plas rv	GCGAATTCGTGGATCCAGATGCCAAAGACGCGACTACAACTA
pNPTS CN-32 *pdeB-gfp* K527S Q528S	TR564 pdeb OL plas fw	GCCAAGCTTCTCTGCAGGATGCAAGGCAATATGGATCCATCC
	TR565	TGATGCTGCTTAACAACTGCACCACAGATAAAGC
	TR566	GCAGTTGTTAAGCAGCATCAGTGCTCAAGTCTCATTACAAG

	TR567 pdeB OL plas rv	GCGAATTCGTGGATCCAGATGCCAAAGACGCGACTACAACTA
pNPTS CN-32 *pdeB-gfp* K527D	TR244 ol pdeB up	GCCAAGCTTCTCTGCAGGATGCAAGGCAATATGGATCCATCC
	TR399	TGATCTGGTCTAACAACTGCACCACAGATAAAGC
	TR400	GGTGCAGTTGTTAGACCAGATCAGTGCTCAAGTCTCATTA
	TR247 rv PdeB down	GCGAATTCGTGGATCCAGATGCCAAAGACGCGACTACAACTA
pNPTS CN-32 *pdeB-gfp* K527D Q528S	TR244 ol pdeB up	GCCAAGCTTCTCTGCAGGATGCAAGGCAATATGGATCCATCC
	TR344 KQ to ES rv	TGATCGAGTCTAACAACTGCACCACAGATAAAGCACTGCGAT
	TR345 KQ to ES fw	GCAGTTGTTAGACTCGATCAGTGCTCAAGTCTCATTACAAG
	TR247 rv PdeB down	GCGAATTCGTGGATCCAGATGCCAAAGACGCGACTACAACTA
pNPTS CN-32 *pdeB-gfp* Q524A K527D Q528A	TR244 ol pdeB up	GCCAAGCTTCTCTGCAGGATGCAAGGCAATATGGATCCATCC
	TR281	TGCGTCTAACAATGCCACCACAGATAAAGCACTGCGA
	TR282	GCATTGTTAGACGCAATCAGTGCTCAAGTCTCATTACAAG
	TR247 rv PdeB down	GCGAATTCGTGGATCCAGATGCCAAAGACGCGACTACAACTA
pNPTS CN-32 *pdeB-gfp* G497A	TR564 pdeb OL plas fw	GCCAAGCTTCTCTGCAGGATGCAAGGCAATATGGATCCATCC
	TR570 G rv	CCTGTGGAGCAAGGCAGGCTTGCATCACTTTAG
	TR571 G fw	AGCCTGCCTTGCTCCACAGGAGTTATTGGGGCGGATTGGTGG
	TR567 pdeB OL plas rv	GCGAATTCGTGGATCCAGATGCCAAAGACGCGACTACAACTA
pNPTS CN-32 *pdeB-gfp* Q499S	TR564 pdeb OL plas fw	GCCAAGCTTCTCTGCAGGATGCAAGGCAATATGGATCCATCC
	TR572 Q rxxd rv	CGCTTGGACCAAGGCAGGCTTGCATCACTTTAG
	TR573 Q rxxd rv	AGCCTGCCTTGGTCCAAGCGAGTTATTGGGGCGGATTGGTGG
	TR567 pdeB OL plas rv	GCGAATTCGTGGATCCAGATGCCAAAGACGCGACTACAACTA
pNPTS CN-32 *pdeB-gfp* E500S	TR564 pdeb OL plas fw	GCCAAGCTTCTCTGCAGGATGCAAGGCAATATGGATCCATCC
	TR574 E rxxd rv	TCTGTGGACCAAGGCAGGCTTGCATCACTTTAG
	TR575 E rxxd rv	AGCCTGCCTTGGTCCACAGAGCTTATTGGGGCGGATTGGTGG
	TR567 pdeB OL plas rv	GCGAATTCGTGGATCCAGATGCCAAAGACGCGACTACAACTA
pNPTS CN-32 *pdeB-gfp* Q524S Q528S	TR244 ol pdeB up	GCCAAGCTTCTCTGCAGGATGCAAGGCAATATGGATCCATCC
	TR347 SLLKS rv	TTAACAAACTCACCACAGATAAAGCACTGCGAT
	TR348 SLLKS fw	ATCTGTGGTGAGTTTGTTAAAGTCGATCAGTGCTCAAGTCTCATTACAAG
	TR247 rv PdeB down	GCGAATTCGTGGATCCAGATGCCAAAGACGCGACTACAACTA
pNPTS CN-32 *pdeB-gfp* K527E Q528S	TR564 pdeb OL plas fw	GCCAAGCTTCTCTGCAGGATGCAAGGCAATATGGATCCATCC
	TR395* ES rv	TGATCGATTCTAACAACTGCACCACAGATAAAGC

	TR396* ES fw	GCAGTTGTTAGAATCGATCAGTGCTCAA GTCTCATTACAAG
	TR567 pdeB OL plas rv	GCGAATTCGTGGATCCAGATGCCAAAG ACGCGACTACAACTA
pNPTS CN-32 *pdeB-gfp* K490D Q493A	TR244 ol pdeB up	GCCAAGCTTCTCTGCAGGATGCAAGGC AATATGGATCCATCC
	TR279	TGCCATCACGTCAGCAACCATGGCCAA CATGC
	TR280	GACGTGATGGCAGCCTGCCTTGGTCCA CAG
	TR247 rv PdeB down	GCGAATTCGTGGATCCAGATGCCAAAG ACGCGACTACAACTA
pNPTS CN-32 *pdeB-gfp* R557G A558G P559G Y560G	TR244 ol pdeB up	GCCAAGCTTCTCTGCAGGATGCAAGGC AATATGGATCCATCC
	TR252	GCCACCCCCTCCACCAAAGGCGACACC GATACTT
	TR253	GGAGGGGGTGGCATCAATGCCCAAGAG TTGTTGAA
	TR247 rv PdeB down	GCGAATTCGTGGATCCAGATGCCAAAG ACGCGACTACAACTA
pNPTS CN-32 *pdeB-gfp* V522G V523G Q524G	TR244 ol pdeB up	GCCAAGCTTCTCTGCAGGATGCAAGGC AATATGGATCCATCC
	TR248	AACCCCCTCCAGATAAAGCACTGCGATT ACAAATCA
	TR249	GGAGGGGGTTTGTTAAAGCAGATCAGT GCTCAAG
	TR247 rv PdeB down	GCGAATTCGTGGATCCAGATGCCAAAG ACGCGACTACAACTA
pNPTS CN-32 *pdeB-gfp* K490G V491G M492G Q593G	TR244 ol pdeB up	GCCAAGCTTCTCTGCAGGATGCAAGGC AATATGGATCCATCC
	TR279	TGCCATCACGTCAGCAACCATGGCCAA CATGC
	TR280	GACGTGATGGCAGCCTGCCTTGGTCCA CAG
	TR247 rv PdeB down	GCGAATTCGTGGATCCAGATGCCAAAG ACGCGACTACAACTA
pNPTS CN-32 *pdeB-gfp* K578S	TR564 pdeb OL plas fw	GCCAAGCTTCTCTGCAGGATGCAAGGC AATATGGATCCATCC
	TR600 cn32 Ksalt to S rv	CGCCCCCTTCGCGCTAGCAGCAAGACA GGCAATATCAG
	TR601 cn32 Ksalt to S fw	GCCTGTCTTGCTGCTAGCGCGAAGGGG GCGAATCAAAT
	TR567 pdeB OL plas rv	GCGAATTCGTGGATCCAGATGCCAAAG ACGCGACTACAACTA
pNPTS CN-32 *pdeB-gfp* K580S	TR564 pdeb OL plas fw	GCCAAGCTTCTCTGCAGGATGCAAGGC AATATGGATCCATCC
	TR602 cn32 Kc to S rv	TTGATTCGCCCCGCTCGCTTTAGCAGCA AGACAGG
	TR603 cn32 Kc to S fw	CTTGCTGCTAAAGCGAGCGGGGCGAAT CAAATCCATATTTATG
	TR567 pdeB OL plas rv	GCGAATTCGTGGATCCAGATGCCAAAG ACGCGACTACAACTA
pNPTS CN-32 *pdeB* G497A	TR564 pdeb OL plas fw	GCCAAGCTTCTCTGCAGGATGCAAGGC AATATGGATCCATCC
	TR570 G rv	CCTGTGGAGCAAGGCAGGCTTGCATCA CTTTAG
	TR571 G fw	AGCCTGCCTTGCTCCACAGGAGTTATTG GGGCGGATTGGTGG
	TR567 pdeB OL plas rv	GCGAATTCGTGGATCCAGATGCCAAAG ACGCGACTACAACTA

pNPTS CN-32 *pdeB* K578S	TR564 pdeb OL plas fw	GCCAAGCTTCTCTGCAGGATGCAAGGCAATATGGATCCATCC
	TR600 cn32 Ksalt to S rv	CGCCCCCTTCGCGCTAGCAGCAAGACAGGCAATATCAG
	TR601 cn32 Ksalt to S fw	GCCTGTCTTGCTGCTAGCGCGAAGGGGGCGAATCAAAT
	TR567 pdeB OL plas rv	GCGAATTCGTGGATCCAGATGCCAAAGACGCGACTACAACTA
pNPTS CN-32 *pdeB* K580S	TR564 pdeb OL plas fw	GCCAAGCTTCTCTGCAGGATGCAAGGCAATATGGATCCATCC
	TR602 cn32 Kc to S rv	TTGATTCGCCCCGCTCGCTTTAGCAGCAAGACAGG
	TR603 cn32 Kc to S fw	CTTGCTGCTAAAGCGAGCGGGGCGAATCAAATCCATATTTATG
	TR567 pdeB OL plas rv	GCGAATTCGTGGATCCAGATGCCAAAGACGCGACTACAACTA
pNPTS CN-32 *pdeB-mvenus*	TR244 ol pdeB up	GCCAAGCTTCTCTGCAGGATGCAAGGCAATATGGATCCATCC
	TR456 PdeB-Venus up rv	CTCGCCCTTGCTCACTGCGCGTTGTGCTAAACCCATCTCA
	TR457 Venus OL PdeB fw	TTAGCACAACGCGCAGTGAGCAAGGGCGAGGAGCTGTTCA
	TR458 Venus OL PdeB fw	AGCGCAAATTCATCACTTGTACAGCTCGTCCATGCCGAGA
	TR459 PdeB-Venus dn fw	GACGAGCTGTACAAGTGATGAATTTGCGCTTTTAGTCCGA
	TR247 rv PdeB down	GCGAATTCGTGGATCCAGATGCCAAAGACGCGACTACAACTA
pNPTS CN-32 *pdeB-venus* D508A E509A	TR244 ol pdeB up	GCCAAGCTTCTCTGCAGGATGCAAGGCAATATGGATCCATCC
	TR456 PdeB-Venus up rv	CTCGCCCTTGCTCACTGCGCGTTGTGCTAAACCCATCTCA
	TR457 Venus OL PdeB fw	TTAGCACAACGCGCAGTGAGCAAGGGCGAGGAGCTGTTCA
	TR458 Venus OL PdeB fw	AGCGCAAATTCATCACTTGTACAGCTCGTCCATGCCGAGA
	TR459 PdeB-Venus dn fw	GACGAGCTGTACAAGTGATGAATTTGCGCTTTTAGTCCGA
	TR247 rv PdeB down	GCGAATTCGTGGATCCAGATGCCAAAGACGCGACTACAACTA
pNPTS CN-32 *pdeB-venus* E637A	TR244 ol pdeB up	GCCAAGCTTCTCTGCAGGATGCAAGGCAATATGGATCCATCC
	TR456 PdeB-Venus up rv	CTCGCCCTTGCTCACTGCGCGTTGTGCTAAACCCATCTCA
	TR457 Venus OL PdeB fw	TTAGCACAACGCGCAGTGAGCAAGGGCGAGGAGCTGTTCA
	TR458 Venus OL PdeB fw	AGCGCAAATTCATCACTTGTACAGCTCGTCCATGCCGAGA
	TR459 PdeB-Venus dn fw	GACGAGCTGTACAAGTGATGAATTTGCGCTTTTAGTCCGA
	TR247 rv PdeB down	GCGAATTCGTGGATCCAGATGCCAAAGACGCGACTACAACTA
pNPTS MR-1 Δ*pdeB*	TR582 SO pdeB KO cterm500	GCCAAGCTTCTCTGCAGGATGCCAAGCCATAATCTTATGCTTTAGG
	TR583 SO pdeB KO start	GTTGTGCTAAGTTGCCTATGCGCATCTTTTACC
	TR584 SO pdeB KO stop	CATAGGCAACTTAGCACAACGCGCATAGGG
	TR581 SO pdeB nterm500	GCGAATTCGTGGATCCAGATTAACAGCATGTTTAGACGCCGC
pNPTS MR-1 *pdeB-gfp*	TR576 SO up fw	GCCAAGCTTCTCTGCAGGATCCGCAGCAGAGCGTTTTAAGC

	TR577 SO pdeb nterm rv	TGCTGCTGCCTGCGCGTTGTGCTAAGCGC
	TR578 SO pdeb-gfp fw	ACAACGCGCAGGCAGCAGCAAAGGAGAAGAACTTTTC
	TR579 SO pdeb-gfp rv	CAATCCCCTAGGATCCTTTGTAGAGCTCATCC
	TR580 SO pdeb nterm fw	CAAAGGATCCTAGGGGATTGCGCTTTTAAGGTG
	TR581 SO pdeB nterm500	GCGAATTCGTGGATCCAGATTAACAGCATGTTTAGACGCCGC
pNPTS MR-1 *pdeB-gfp* K524E Q525S	TR582 SO pdeB KO cterm500	GCCAAGCTTCTCTGCAGGATGCCAAGCCATAATCTTATGCTTTAGG
	TR593 SO KQ to ES rv	TAATGCTTTCCAGCAACTGCGCCACAGCTAA
	TR594 SO KQ to ES fw	GCAGTTGCTGGAAAGCATTATTGCTCAAGTATCGCTGCAAGTG
	TR581 SO pdeB nterm500	GCGAATTCGTGGATCCAGATTAACAGCATGTTTAGACGCCGC
pNPTS MR-1 *pdeB-gfp* G494A	TR582 SO pdeB KO cterm500	GCCAAGCTTCTCTGCAGGATGCCAAGCCATAATCTTATGCTTTAGG
	TR544 G494A fw	ATTCCTGTGGCGCAAGACATGACTGAATCGCCCTAG
	TR555 G494A rv	ATGTCTTGCGCCACAGGAATTATTAGCCCGCA
	TR581 SO pdeB nterm500	GCGAATTCGTGGATCCAGATTAACAGCATGTTTAGACGCCGC
pNPTS MR-1 *pdeB-gfp* K575S	TR582 SO pdeB KO cterm500	GCCAAGCTTCTCTGCAGGATGCCAAGCCATAATCTTATGCTTTAGG
	TR598 aSak rv	GGTGCCCTTGGCACTAGCGGCAATACAGGCGATATCT
	TR599 aSak fw	GCCTGTATTGCCGCTAGTGCCAAGGGCACCAACCAAAT
	TR581 SO pdeB nterm500	GCGAATTCGTGGATCCAGATTAACAGCATGTTTAGACGCCGC
pNPTS MR-1 *pdeB-gfp* K577S	TR582 SO pdeB KO cterm500	GCCAAGCTTCTCTGCAGGATGCCAAGCCATAATCTTATGCTTTAGG
	TR596 akaS rv	TTGGTTGGTGCCACTGGCTTTAGCGGCAATACAGG
	TR597 akaS fw	ATTGCCGCTAAAGCCAGTGGCACCAACCAAATCCACATTTA
	TR581 SO pdeB nterm500	GCGAATTCGTGGATCCAGATTAACAGCATGTTTAGACGCCGC

Supplementary References

1 Miller, V. L. & Mekalanos, J. J. A novel suicide vector and its use in construction of insertion mutations: Osmoregulation of outer membrane proteins and virulence determinants in *Vibrio cholera* requires *toxR*. *J. Bacteriol.***170**, 2575–2583 (1988).

2 kindly provided by William Metcalf, University of Illinois, Urbana-Champaign.

3 New England Biolabs, Ipswich, MA

4 Fredrickson, J. K. et al. Biogenic iron mineralization accompanying the dissimilatory reduction of hydrous ferric oxide by a groundwater bacterium. *Geochim. Cosmochim. Acta* **62**, 3239–3257 (1998).

5 Bubendorfer, S., Koltai, M., Rossmann, F., Sourjik, V. & Thormann, K. M. Secondary bacterial flagellar system improves bacterial spreading by increasing the directional persistence of swimming. *Proc. Natl. Acad. Sci. U. S. A.* **111**, 11485–11490 (2014).

6 Rossmann, F.M. et al. The GGDEF domain of the phosphodiesterase PdeB in *Shewanella putrefaciens* mediates recruitment by the polar landmark protein HubP. *J Bacteriol.* **201**, 7 e00534-18. (2019).

7 kindly provided by Fitnat Yildiz, UC Santa Cruz, CA.

8 Venkateswaran, K. et al. Polyphasic taxonomy of the genus *Shewanella* and description of *Shewanella oneidensis* sp. nov. *Int. J. Syst. Evol. Microbiol*. **49**, 705–724 (1999).

9 Lassak, J., Henche, A. L., Binnenkade, L. & Thormann, K. M. ArcS, the cognate sensor kinase in an atypical arc system of *Shewanella oneidensis* MR-1. *Appl. Environ. Microbiol*. **76**, 3263–3274 (2010).

10 EMD Biosciences, Merck KGAA, Darmstadt, Germany

11 Rossmann, F. et al. The role of FlhF and HubP as polar landmark proteins in *Shewanella putrefaciens* CN-32. *Mol. Microbiol.* **98**, 727–742 (2015).

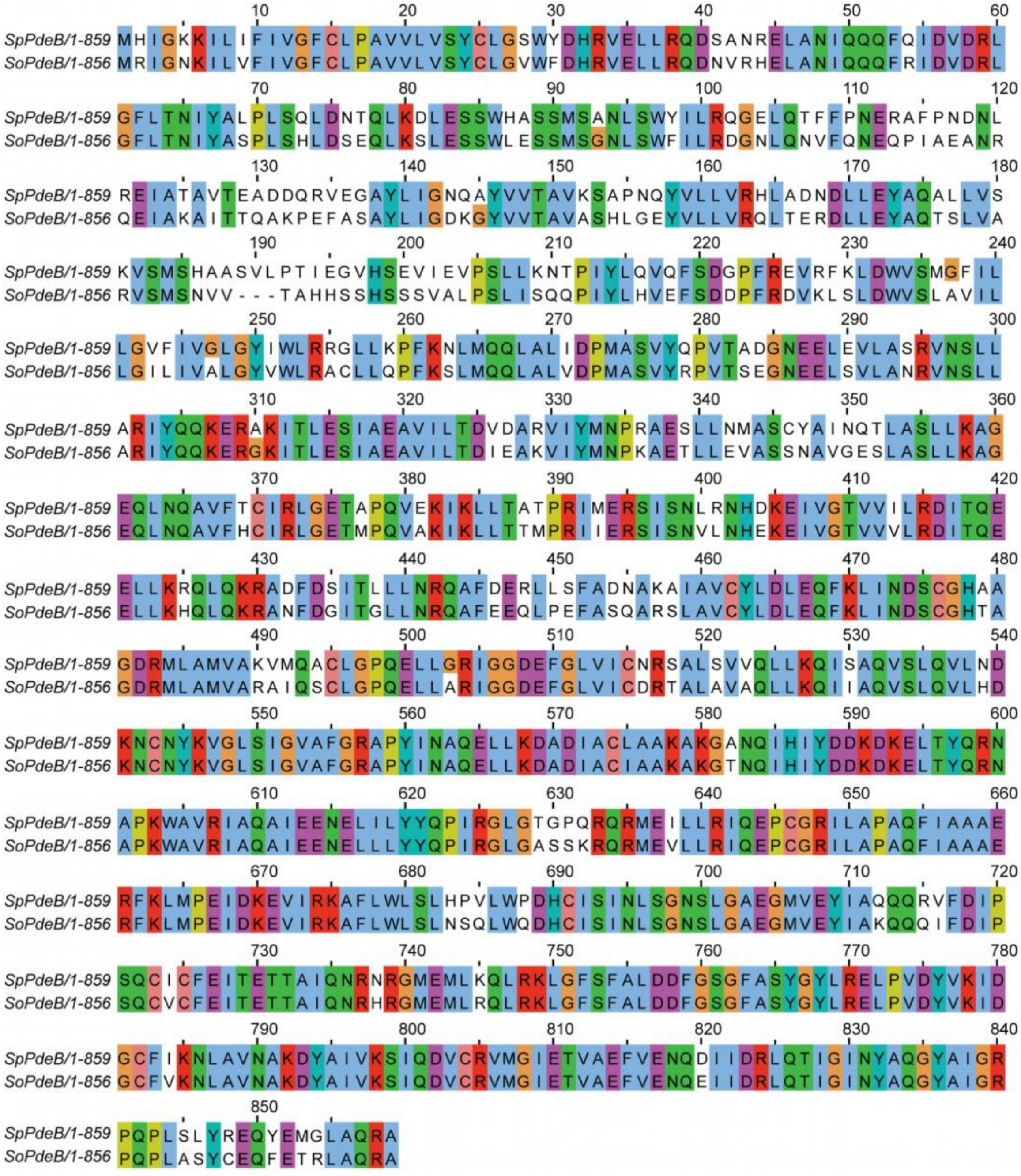

Supplementary Figure 1: Alignment of *Sp*PdeB with *So*PdeB. The conservation between PdeB homologous of *S. putrefaciens* and *S. oneidensis* was analyzed by aligning the amino acid sequences. The two proteins share 79% identity. Colored residues indicate 100% conservation between the two proteins. Periplasmic region: 30 - 230; HAMP: 255 - 308; PAS: 308 - 376; GGDEF: 420 - 588; EAL: 598 - 846.

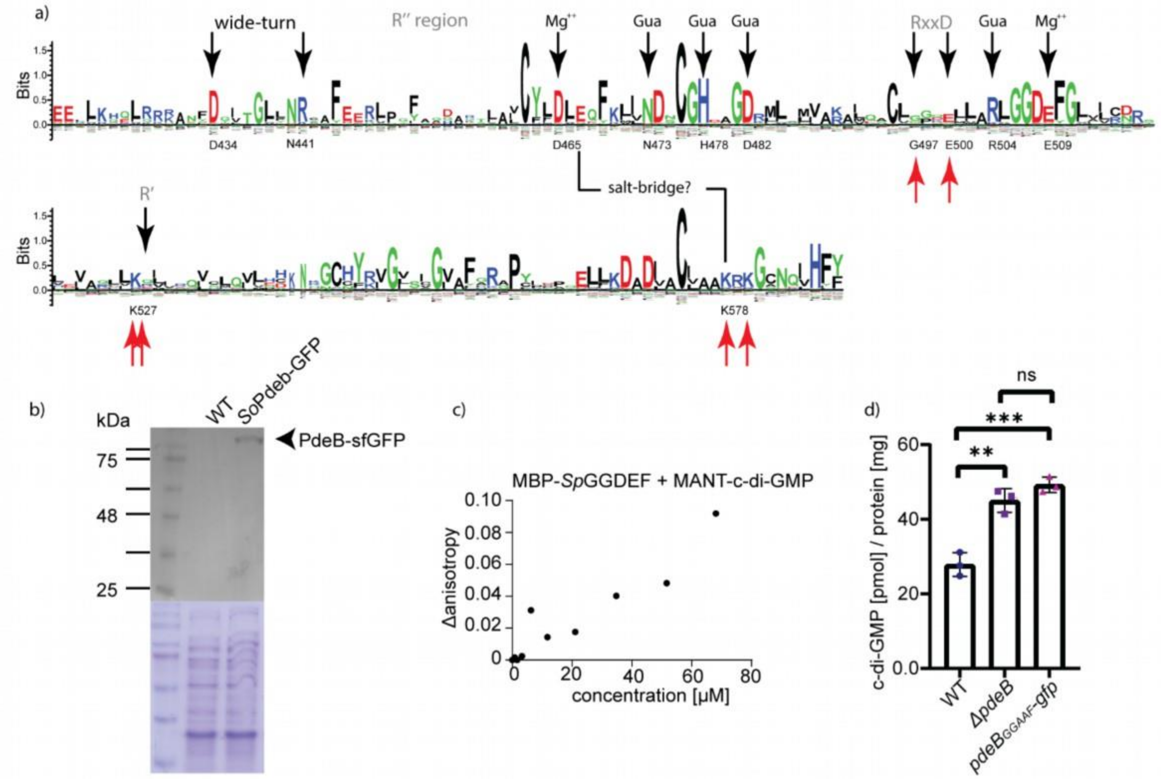

Supplementary Figure 2. a) Position-based weight map of the GGDEF domain of 50 PdeB homologues from different *Shewanella* species. Characteristic features of GGDEF domains are marked with black arrows, and degenerated or missing motifs are indicated in gray. Residues that are important for the $GGDEF_{PdeB}$-$FimVc_{HubP}$ interaction are highlighted by red arrows. **b)** The stability and expression of genomic *So*PdeB-sfGFP fusions was verified by immunoblot analysis. **c)** The MANT-c-di-GMP binding of the GGDEF domain of *So*PdeB was tested by fluorescence anisotropy assays. No binding curve was observed, but only unspecific binding at unphysiological high ligand concentrations. **d)** The effect of GTP binding to $GGDEF_{PdeB}$ on the PDE activity of PdeB was determined by introducing mutations the GGDEF motif. The cellular c-di-GMP content was then extracted and quantified by MS. The single point mutation within the GGDEF motif results in a similar increase in cellular c-di-GMP as deletion of *pdeB*. Significance was tested by using the unpaired t-test (* $P < 0.05$, ** $P < 0.005$, *** $P < 0.0005$).

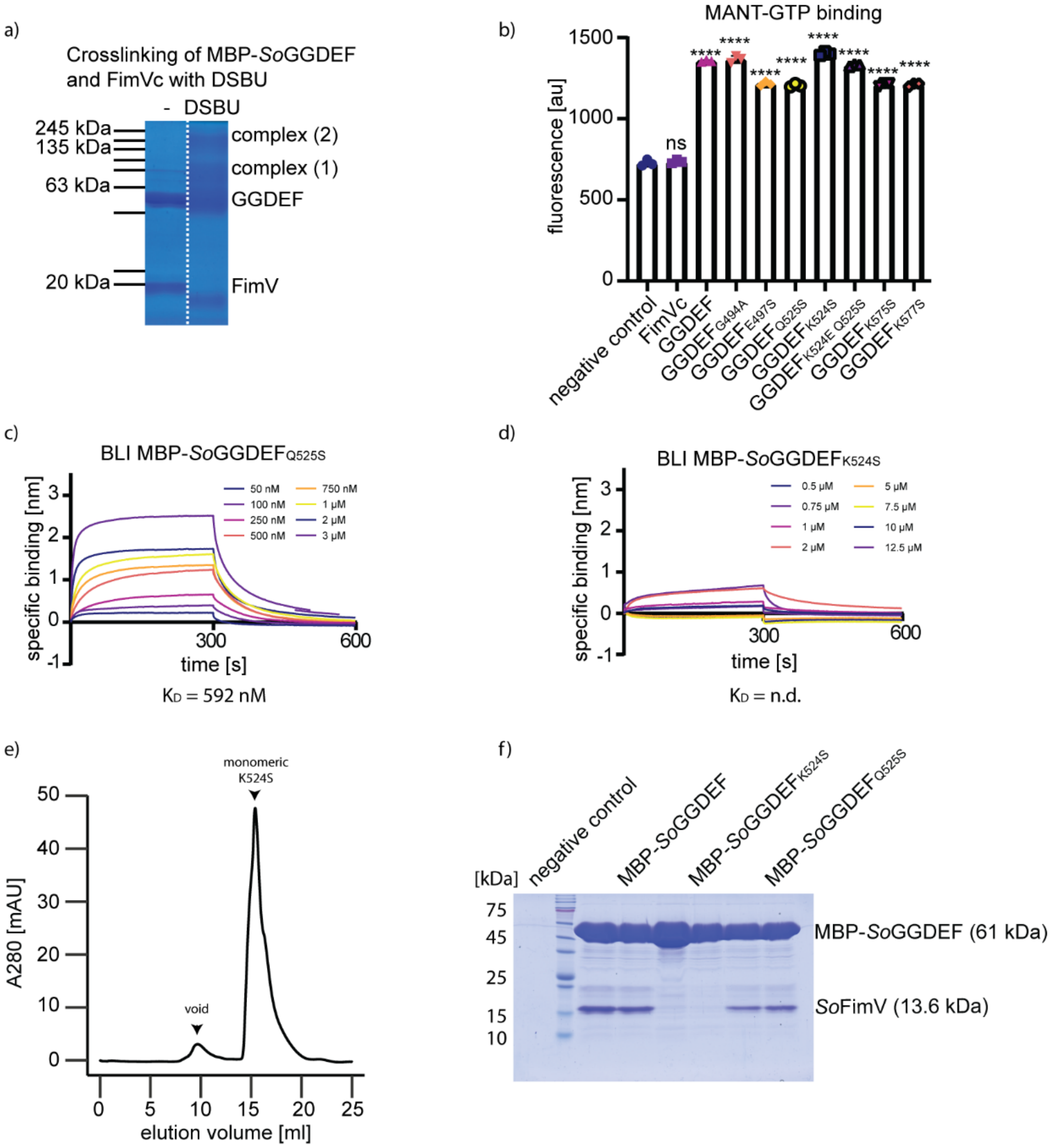

Supplementary Figure 3: Structural and sequential features of PdeB$_{GGDEF}$ and HubP$_{FimV}$. a) The crosslinking of *So*GGDEF with *So*HubP was verified by SDS-PAGE. The unprocessed PAGE can be found in Supplementary Figure 11. **b)** Functionality of MBP-*So*GGDEF proteins used for BLI was shown by MANT-GTP binding assays. All mutated versions are able to bind MANT-GTP, as indicated by the increased fluorescence. Significance was tested by using the unpaired t-test (* $P < 0.05$, ** $P < 0.005$, *** $P < 0.0005$, **** $P < 0.00005$). **c, d)** BLI assays for GGDEF proteins with substitutions in the R` I-site show decreased affinity to FimV compared to the wild type. The purified MBP-GGDEF$_{K524S}$ showed aggregation upon production and unspecific binding in BLI assays and was therefore not suitable for determination of exact KD values. **e)** The stability was therefore tested by storing the protein for three days at 4°C and further analysed with SEC. The sample remained mostly in monomeric form. **f)** The interaction of MBP-GGDEF$_{K524S}$ with FimV was tested by pull-down assays where the wild-type version and MBP-GGDEF$_{Q525S}$ served as controls. No binding was observed when K524 was mutated.

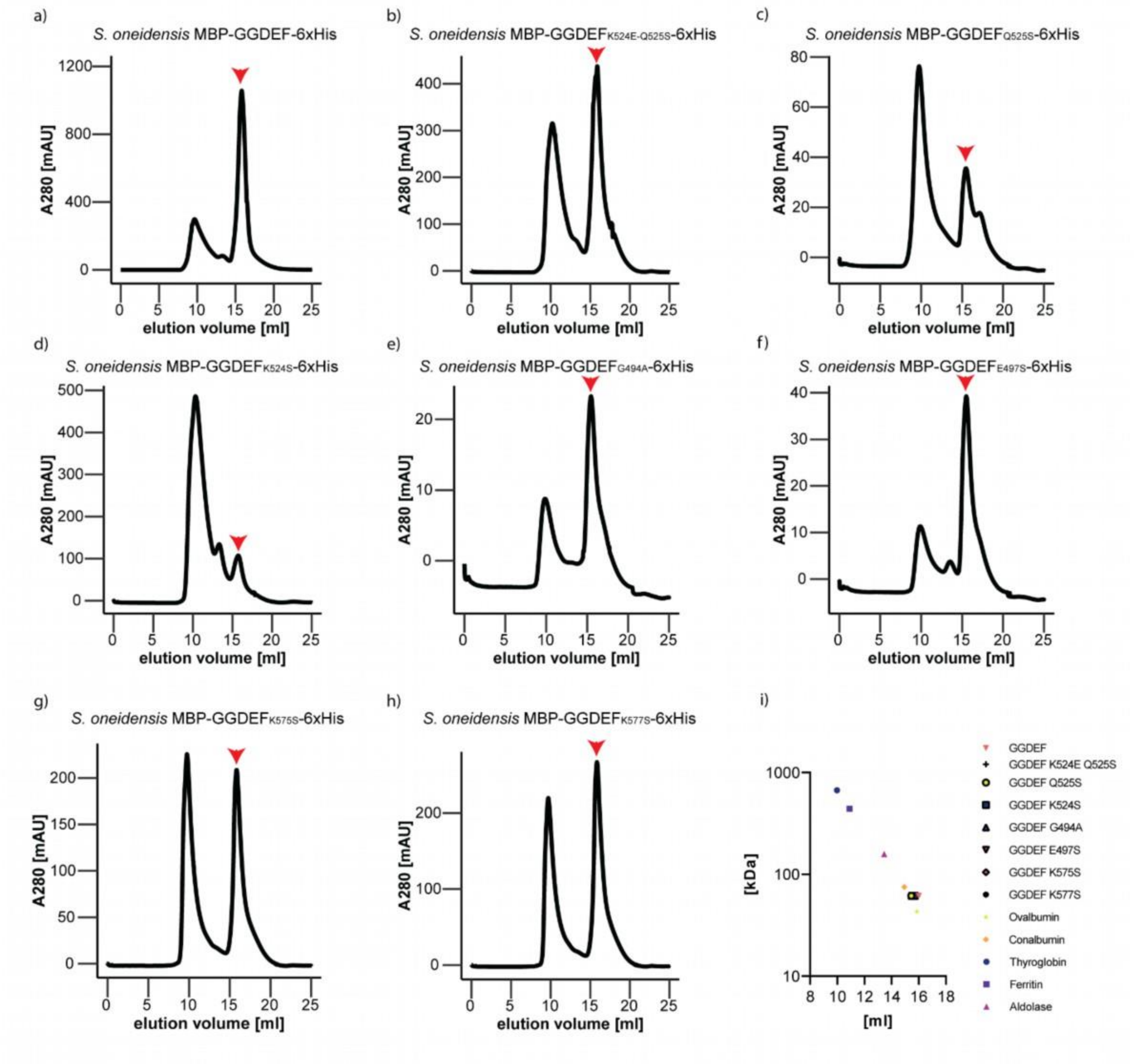

Supplementary Figure 4: SEC of GGDEF domains. a-h) The mutated $GGDEF_{PdeB}$ domains of *S. oneidensis* were purified as MBP-fusion proteins. The chromatograms are shown in a-h. The elution volume in ml is plotted against the absorbance at 280 nm. Red arrows indicate the peak for the monomeric proteins of interest. **i)** The elution volume of the proteins of interests was plotted against the molecular weight in kDa, together with globular proteins included in the high molecular weight calibration kit (GE healthcare). All GGDEF proteins elute at roughly the same elution volume, indicating that the introduced amino acid substitutions do not alter the mutant proteins' structure.

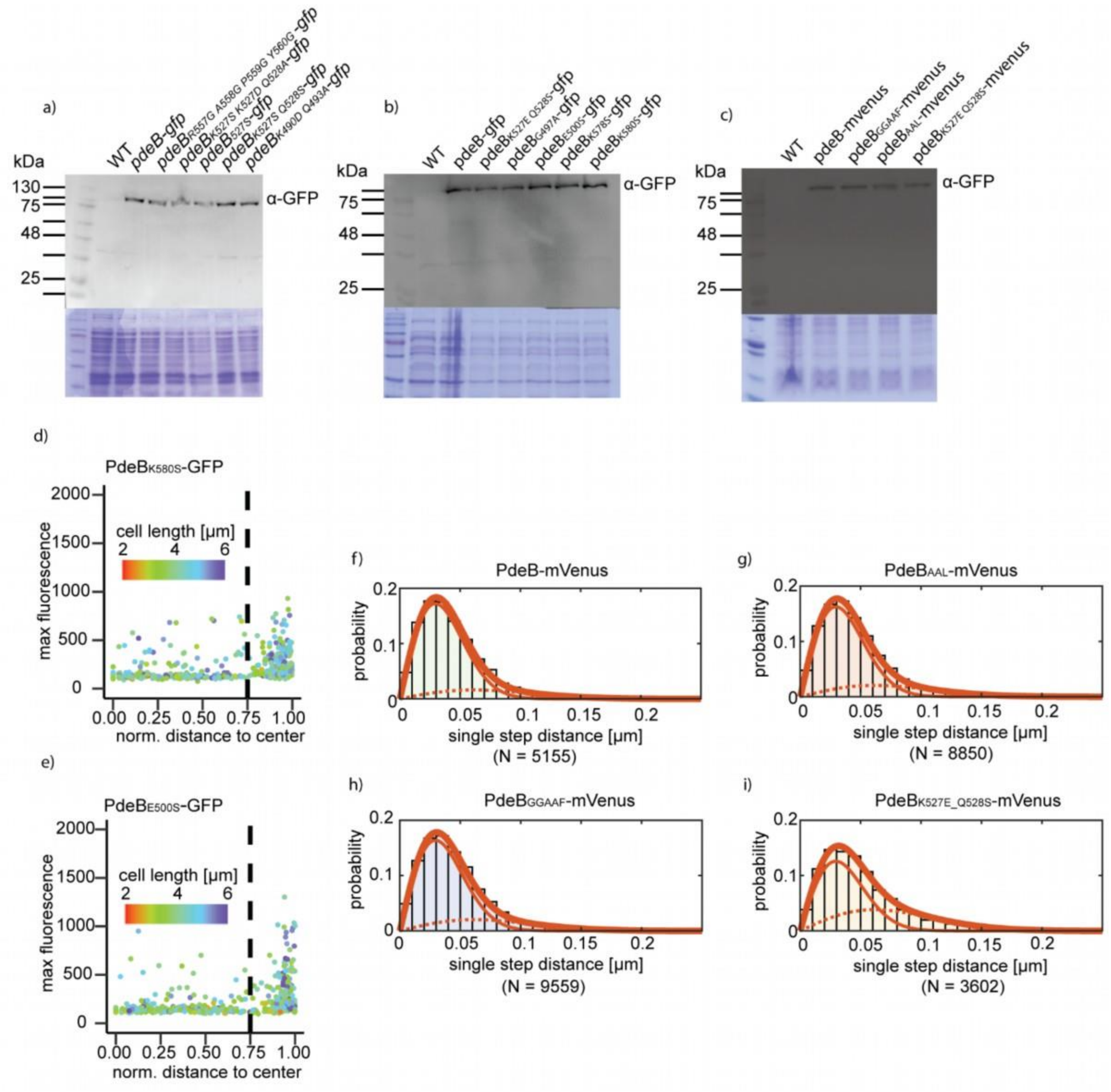

Supplementary Figure 5. a-b) Stability and expression of *Sp*PdeB-sfGFP mutants were verified using Western blot analysis. **c)** Stability of *Sp*PdeB-mVenus used for single molecule microscopy was also shown by Western blot analysis. **d-e)** Scatterplots of fluorescence microscopy of mutants with reduced PdeB-sfGFP localization, displaying the normalized distance from the central plane of the cell. Both mutations reduce the polar localization of PdeB-sfGFP, likely due to reduced affinity to HubP. **f-i)** Fits for the jump distance analyses used for the data of the bubble blot. Solid thin line Rayleigh fit for the slow population, dotted line fast population, and thick solid line combination of both fits. R^2 of values all fits were higher than 0.999. For all mVenus fusions, a two population-fit was the best.

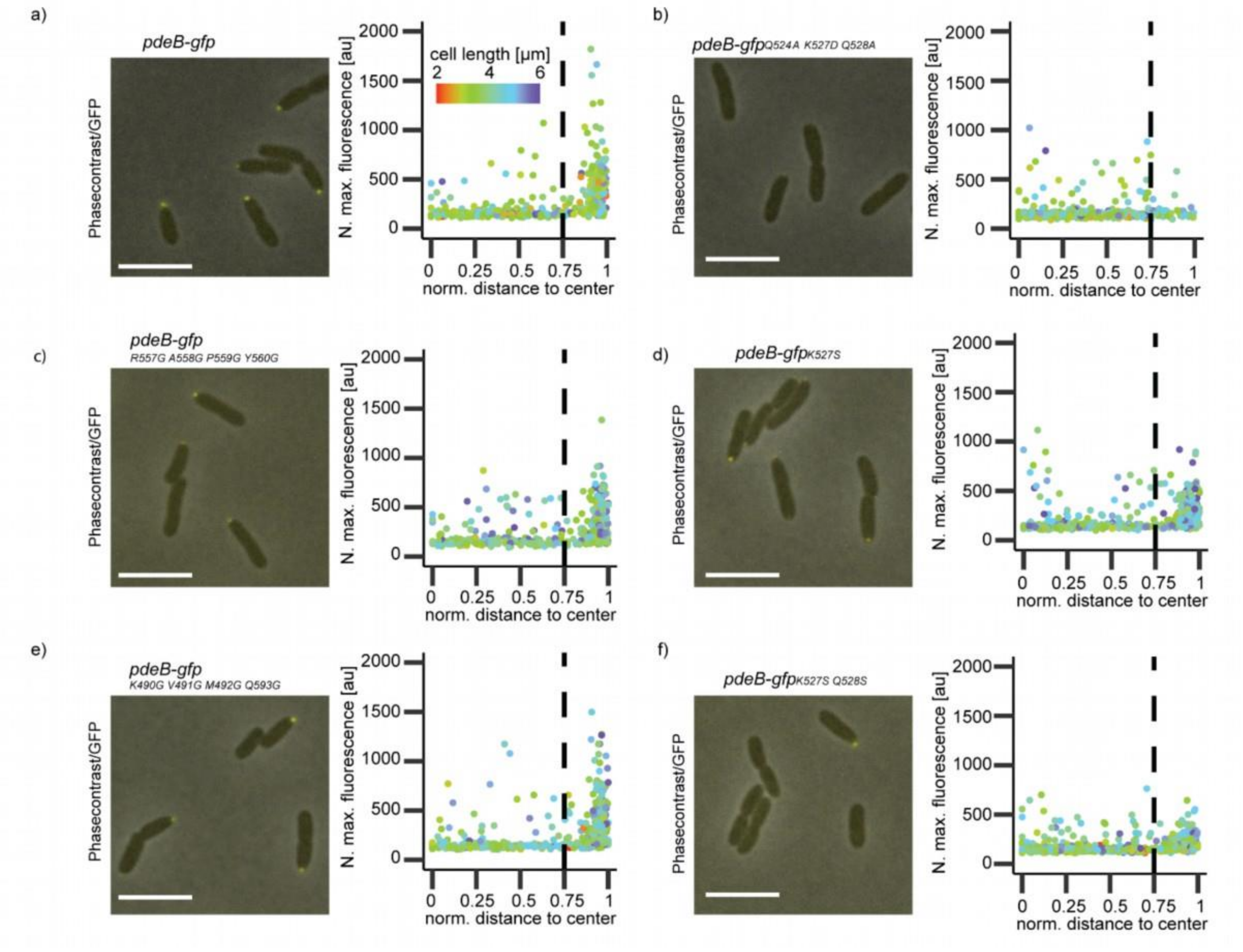

Supplementary Figure 6: Screening for residues involved in the polar localization of PdeB. **a-f)** Residues at different regions in the GGDEF domain of *Sp*PdeB-sfGFP were genomically mutated and localization behavior was observed using fluorescence microscopy. The localization behaviors of mutants are shown as scatter plots displaying the normalized distance from the central plane of the cell, wild-type *Sp*PdeB-sfGFP serves as control. Mutating residues of the R` site (b, d, f) leads to reduced polar localization, while substitutions in the other two regions only had minor effects.

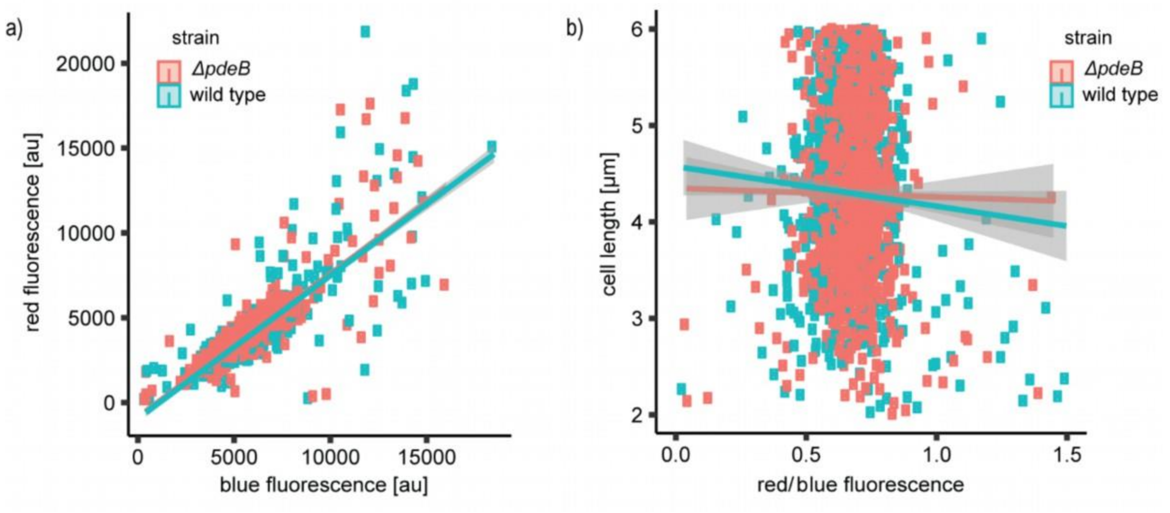

Supplementary Figure 7: c-di-GMP single cell reporter controls. a) The functionality of the fluorescence based c-di-GMP reporter was tested for *S. putrefaciens* CN-32 by plotting the blue against the red fluorescence and testing for linear correlation in presence and absence of *pdeB*. **b)** A correlation of cell length with the c-di-GMP level was tested by plotting the quotient of the red fluorescence divided by the blue fluorescence against the cell length. No such correlation was found.

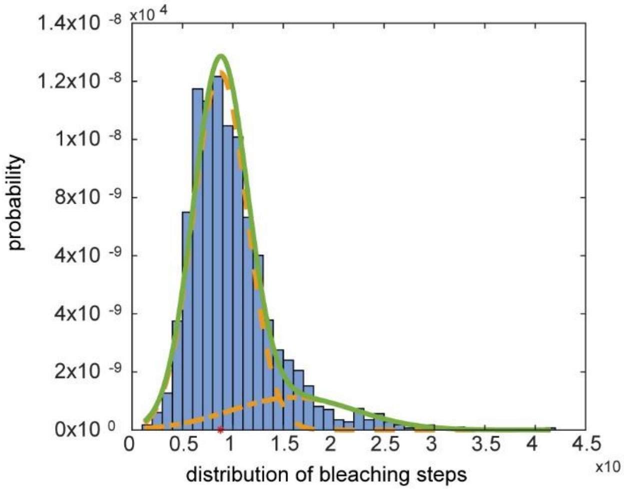

Supplementary Figure 8: Fluorescence-based molecule quantification of PdeB-mVenus. The distribution of bleaching steps within the movies is shown as histogram.

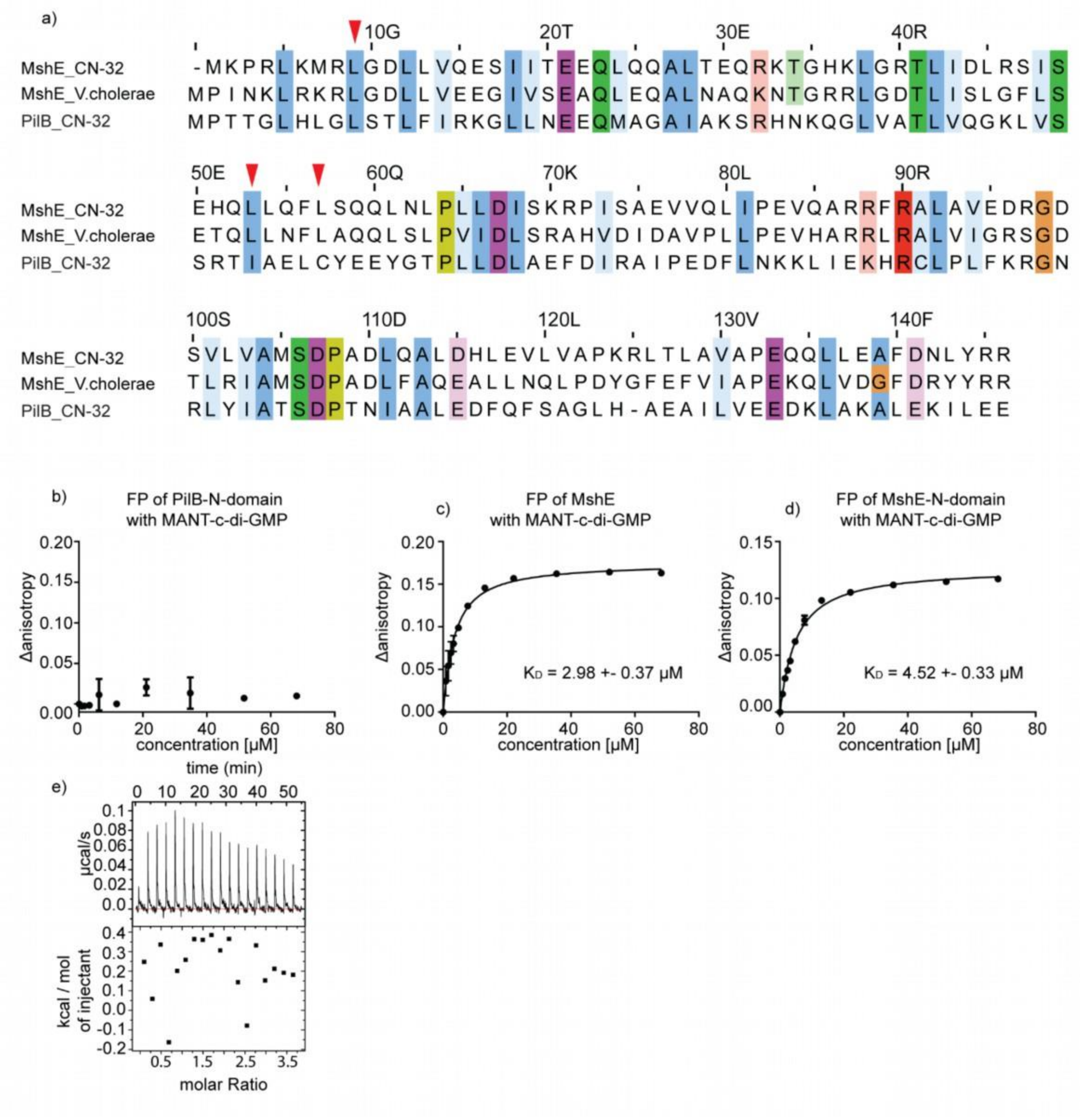

Supplementary Figure 9. a) Alignment of *Pa*MshE with *Sp*MshE and *Sp*PilB. Leucines that are involved in c-di-GMP binding are marked with red arrows. **b-d)** The MANT-c-di-GMP binding of the N-terminal domain of PilB (b), MshE (c) and the N-terminal domain of MshE (d) was tested by fluorescence anisotropy measurements. Both MshE versions are able to bind MANT-c-di-GMP with high affinity, while PilB does not. **e)** The non-binding of c-di-GMP to PilB was verified by ITC.

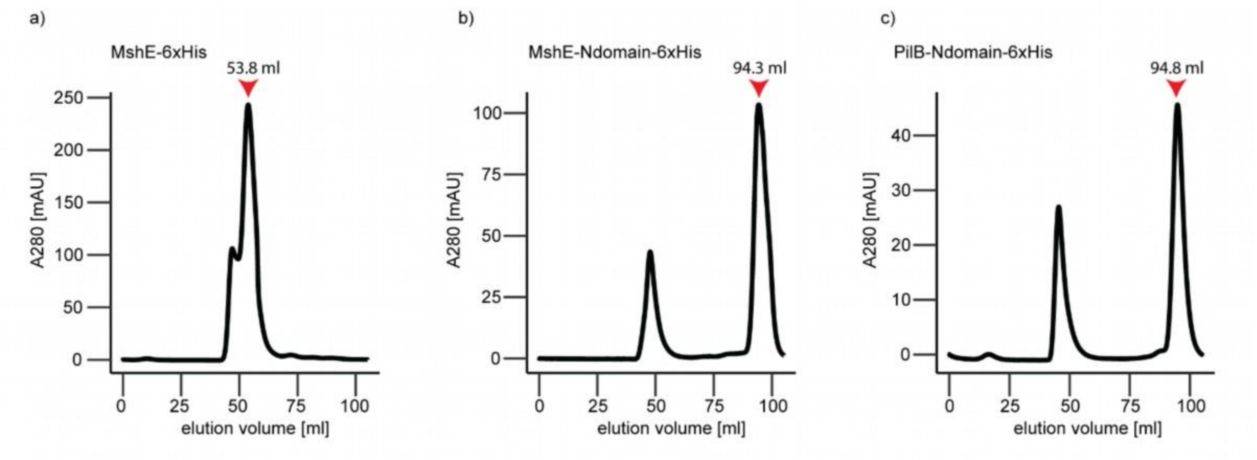

Supplementary Figure 10: SEC of MshE and PilB. a-b) Chromatograms of the size-exclusion chromatography of the extension ATPase MshE. Chromatograms show the elution volume against the absorbance at 280 nm. Peaks that contain the protein of interest are indicated by red arrows. The full length MshE protein (a) eluted at 53.8 ml, indicating an oligomeric state (penta- or hexameric), while the N-terminal domain eluted as monomer. **c)** The N-terminal domain of PilB eluted as monomer.

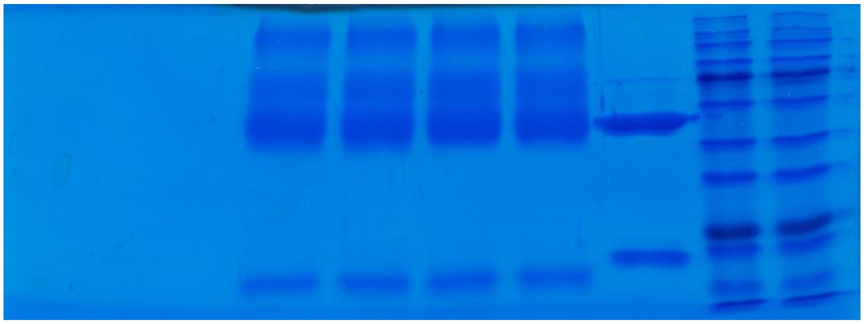

Supplementary Figure 11: Uncropped scan of the crosslinking control PAGE shown in **Supplementary Figure 3a**. The first four panels from the left show different crosslinking preparations, followed by a control of non-crosslinked protein and two protein ladder standards to the right.

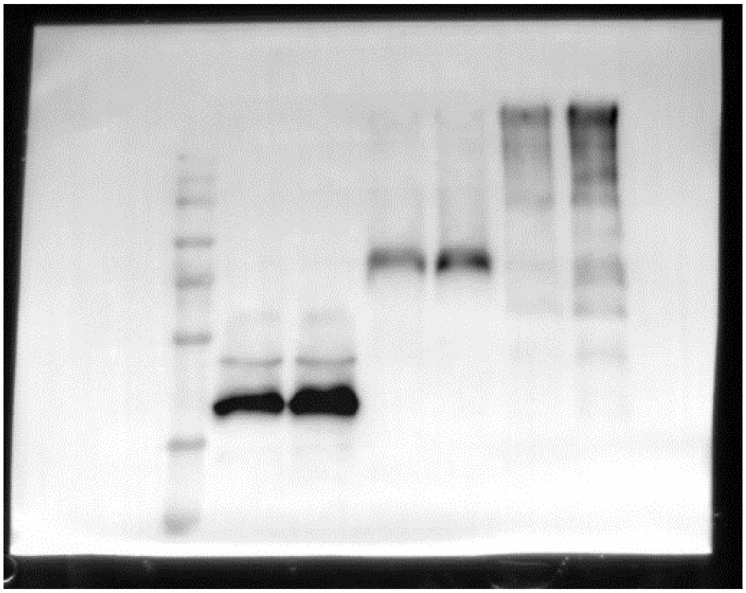

Supplementary Figure 12: Uncropped Western Blot shown in **Figure 6f**. The proteins in lane 2 and 3 right next to the protein standard belong to a different protein and were therefore removed.
